# Predictors of Knowledge, Attitude, and Practice of Kangaroo Mother Care Among Mothers in a Ghanaian Tertiary Care Center

**DOI:** 10.1155/ijpe/9420090

**Published:** 2025-09-03

**Authors:** David Adedia, Felix Kwasi Nyande, Anthony Kuug, Agani Afaya, Martin Amogre Ayanore, Mildred Yibile, Evelyn Tangtie, Linda Cudjoe, Lillie Akanlie Baba Musah, Tackie-Ankrah Beatrice, Magdalene Kunje, Josephine Nana Ama Moreax, Rita Obeng, Francisca Gyan, Efua Essilfua Anaman, Kennedy Diema Konlan

**Affiliations:** ^1^Department of Basic Sciences, School of Basic and Biomedical Sciences, University of Health and Allied Sciences, Ho, Ghana; ^2^Department of Nursing, School of Nursing and Midwifery, University of Health and Allied Sciences, Ho, Ghana; ^3^Department of Health Policy Planning and Management, Fred N. Binka School of Public Health, University of Health and Allied Sciences, Ho, Ghana; ^4^Department of Midwifery, School of Nursing and Midwifery, University of Health and Allied Sciences, Ho, Ghana; ^5^Department of Public Health Nursing, School of Nursing and Midwifery, University of Health and Allied Sciences, Ho, Ghana

**Keywords:** care, infants, kangaroo mother care, modeling, mother, neonates, path analysis

## Abstract

**Introduction:** Kangaroo mother care (KMC) is one of the cost-effective interventions in low-resource settings for effective thermoregulation, supportive breastfeeding, and ensuring early hospital discharge of preterm and low birth weight babies. This study described the predictors of knowledge, attitude, and practice of KMC in a Tertiary Care Center in Ghana.

**Methods:** A cross-sectional design using an online survey was conducted. The convenience sampling method was employed to select 385 mothers. Logistic regression models and path models were used to determine the factors influencing the knowledge, attitude, and practice of KMC.

**Results:** The factors that predict a woman's knowledge of KMC are birthing a preterm or low birth weight baby, aged above 35 years, being a Christian, having health insurance, and giving birth at a health facility. The attitude of mothers toward KMC practice was significantly associated with education, ethnicity, health insurance, weight of newborn, and antenatal clinic (ANC) follow-up. Mothers who used the spontaneous vaginal delivery (SVD) type (aOR = 0.06, 95% CI: 0.01–0.28, *p* value = 0.001) are less likely to practice KMC than mothers who used the caesarean section (C/S) delivery type; however, mothers who used the SVD type and had health insurance (aOR = 16.02, 95% CI: 3.13–94.95, *p* value = 0.001) were more likely to practice KMC. Also, mothers who delivered at a private hospital (aOR = 0.42, 95% CI: 0.18–0.97, *p* value = 0.039) and newborns with weights 1000–1499 g (aOR = 0.32, 95% CI: 0.13–0.72, *p* value = 0.008) and 2000–2500 g (aOR = 0.31, 95% CI: 0.13–0.70, *p* value = 0.006) were less likely to practice KMC. In addition, mothers who are not married (aOR = 1.93, 95% CI: 1.10–3.49, *p* value = 0.025) are more likely to practice KMC.

**Conclusion:** Given that numerous factors influence practice (SVD, having health insurance, not birthing in a private facility, and having a normal birth weight baby) of KMC, promoting health insurance registration, increasing pregnancy safety leading to SVD, and fostering normal birth weight births can limit the need to use KMC. However, given the positive benefits of KMC, ensuring a positive attitude among community members is crucial to its adoption, should it be needed.

## 1. Introduction

Prematurity and low birth weight are among the leading causes of neonatal deaths globally [1, 2], and interventions to promote survival are warranted. During the neonatal period, interventions must ensure adequate thermoregulation, prevent infections, and promote adequate breastfeeding with good mother–baby bonding [3, 4]. Kangaroo mother care (KMC) is one of the cost-effective interventions in low-resource settings for successful thermoregulation, supportive breastfeeding, and ensuring early hospital discharge of preterm and low birth weight babies [4–6]. The use of KMC initiatives in both resourced and resource-limited settings has helped to reduce infant morbidity and mortality, ensured neonatal survival, and has a comparative advantage over the conventional methods of care [5, 7]. Conventional hospital-based care usually appears costly, needs sophisticated equipment, requires a trained service provider, and may not incorporate cultural context [5, 7–9]. This is because the KMC initiative requires the continued contact of the caregiver with the baby, allowing for facilitative monitoring and effective bonding [7, 10].

KMC is one of the effective, easy, culturally friendly, and emotionally supportive baby care methods that allow for direct skin-to-skin contact between the mother/caregiver and the baby [7, 10]. Also, KMC is usually adopted in resource-limited settings where equipment and supportive facilities may not allow for continuous thermoregulation [10]. The adoption of KMC in Africa has revolutionized the care of babies because of the direct involvement of the parent, limiting the risk of physical and emotional injury, reducing hospital admission time, promoting a sense of involvement, facilitating communication with the baby, and the eventual acceptance of the babies by the parents [10]. This care method is natural and limits the social, economic, and physiological risk to the baby and mother [7, 8, 10]. Compared to the incubator-based care of babies, KMC is regarded as one of the methods that reduce the incidence of infection (including neonatal sepsis), nosocomial infection, hypothermia, severe morbidity, lower respiratory tract infections, and prolonged hospital stay [8, 9, 11, 12]. KMC methods can lead to increased body weight and length, improved head circumferences, adequate breathing, and effective breastfeeding [4, 6, 8, 9, 13]. The method is also a culturally sensitive method that incorporates various dimensions of cultures, facilitating its acceptance [8, 14, 15].

Newborn and neonatal deaths globally, especially in Africa, remain considerably high despite the critical reductions in all under-five deaths [13]. Most of all, neonatal deaths can be associated with preterm and low birth weight [13, 16]. Given the positive impact of KMC on the baby and the family, mothers' knowledge is critical in its eventual adoption. Mothers who were trained or received education on KMC during antenatal care (ANC) had a higher chance of adopting the same after birth [8]. The WHO recommends using KMC in resource-limited settings for children under 2000 g, but its direct implementation is still limited. In Ghana, although it is known that newborn deaths can be drastically reduced by adopting KMC practices, their complete and sustained utilization is still limited. It is important that interventions specifically address the challenges associated with this adoption and sustained use. To achieve this, assessing the knowledge, attitude, and practices toward KMC among mothers is warranted. Also, to facilitate its complete adoption, it is critical to identify the factors influencing (barriers and facilitators) KMC in resource-limited settings, especially in sub-Saharan Africa [15]. This study described the predictors of knowledge, attitude, and practice of KMC in a Tertiary Care Center, Ghana. Determining these factors will promote the institutionalization of intervention to encourage adoption and the elimination of barriers.

## 2. Methods

### 2.1. Design

A cross-sectional design was used to obtain an all-encompassing insight into maternal knowledge, attitudes, and implementation of KMC within the neonatal wards at the Ho Teaching Hospital.

### 2.2. Study Population and Sample

The study population consists of postnatal mothers who have recently given birth to a preterm or low birth weight infant and are currently staying in the neonatal wards of the Ho Teaching Hospital. These mothers are directly relevant to the study's focus on KMC and its implementation in the neonatal wards. Participants were required to be 18 years of age or older. This age restriction was aimed at ensuring that the participants had the legal capacity to provide informed consent. Mothers who had mental illness or were not psychologically stable enough to provide coherent responses were not included. Additionally, mothers who were admitted for various reasons after the birth of the baby were also excluded.

Applying Cochran's formula, a sample of 385 was determined based on a 5% significance level, a margin of error of 0.05, and a proportion of 50% was chosen to get a larger sample size. The proportion of 50% prevalence was chosen because of the relative lack of a priori information on the prevalence of KMC use or low birth or preterm birth in the study settings. The convenience sampling method was used to select participants. This deliberate approach involves the careful and intentional choice of postnatal mothers at Ho Teaching Hospital who were required to be actively engaged in the practice of KMC. As a convenience sampling method only allows those who are approached and accept to be included in the study, there was no need to consider possible nonresponse.

### 2.3. Data Collection and Management

The questionnaire was used in collecting the data. The questionnaire was adopted from previous studies [17, 18] that assessed the knowledge, attitude, and practice of KMC in Nigeria. The Likert scale format was adopted, ranging from “strongly disagree” [1] to “strongly agree” [5]. The questionnaire was structured into five distinct sections. Section A focuses on gathering demographic information from the participants. The demographic characteristics that were assessed included age (categorized into those below and above 35 years), marital status (grouped into two, i.e., married and not married), educational status (below and above minimum tertiary level education), ethnicity (as Akan, Ewe, and other tribes included Dagomba, Hausa, Frafra, and Guan), religion (grouped as Christian and non-Christian), employment status (as employed or not employed), wealth index (based on family income level as poor, middle, and rich), possession of valid health insurance, sex of baby, perceived health status of baby (grouped as heathy or sick), mode of delivery of baby (spontaneous vaginal delivery [SVD] and caesarean section [CS]), place of delivery (grouped as government hospital, health center, private hospital), weight of baby at birth, had regular ANC attendance, and the perceived newborn that required the KMC (grouped as preterm or post term/baby greater than 4 kg).

Section B is dedicated to postnatal mothers' utilization of KMC. Section C asked questions on the knowledge of postnatal mothers on KMC, Section D asked questions on the attitudes of mothers toward KMC utilization, and Section E asked questions on barriers influencing the implementation of KMC.

The practice of KMC, knowledge, and attitude of postnatal mothers were the outcome variables. Measuring the practice of KMC quantitatively involves assessing specific behaviors and actions related to KMC and assigning numerical values. Therefore, behavioral indicators were used to determine the practices of KMC with the aid of structured questionnaires. These encompass activities such as direct skin-to-skin contact, the frequency and duration of KMC sessions, proper positioning of the baby on the mother's chest, and breastfeeding frequency. Knowledge of KMC was determined using 14 items on a 5-point Likert scale. Sample questions include “babies who are given KMC cry less,” “KMC promotes babies' growth and development,” and “KMC results in reduced infection in the baby.” Attitude toward KMC was determined using 6 items on a 5-point Likert scale. Sample questions include “I was not scared that the baby might get suffocated during KMC,” “I was not anxious during KMC,” and “I do not see KMC to be tiring.” Barriers to KMC practice were measured on 12 items on a 5-point Likert scale. Sample questions include “I feel the reluctance to initiate KMC,” “I have the fear of accidental extubating,” and “I have the fear of vascular access dislodgement.”

The scales employed in this study showed acceptable reliability with Cronbach's alpha values of 0.6, 0.8, 0.6, and 0.8 for KMC practice, knowledge of KMC, attitude toward KMC, and barriers to KMC practice, respectively. A Cronbach's alpha of at least 0.7 is usually recommended; however, slightly lower values of 0.6 were used in some studies [19, 20], especially when scales were pretested and standardized.

### 2.4. Data Handling and Analysis

The data collection involved Google Forms, which were distributed and completed online through trained research assistants. Respondents were postnatal mothers in the neonatal wards of Ho Teaching Hospital. The data collection commenced in January 2024 till July 2024. This approach is chosen for its ease of distribution, user-friendly interface, and the potential to gather responses efficiently. The survey responses were collated using Microsoft Excel. The data was cleaned in Microsoft Excel and transferred to the Statistical Package for Social Sciences (SPSS) Version 25 for analysis.

Composite scores were determined for each outcome variable and converted to percentages. A mother was classified as having a good attitude, knowledge, or practice with a percentage score of more than 75%. The raw composite scores were used for the path analysis, while the dichotomized data were used in the logistic regression modeling. Agree and highly agree scores for barriers were recoded as agree, while neutral, disagree, and highly disagree were coded as disagree.

Descriptive results were presented as frequencies and percentages. The chi-square test was used as a bivariate method to assess the association between the outcome variables and possible factors.

After the association tests, variables whose *p values* were at most 0.2 were included in the logistic regression models while controlling for age. This was done to include only variables that have a higher chance of contributing to the model and reducing redundancy. The logistic regression models were fitted for knowledge, attitude, and practice of KMC. Three models were fitted to ascertain the predictors of KMC practice among mothers. The first model was fitted with all possible factors of KMC practice that reported *p* values ≤ 0.2 from the bivariate chi-square test of association while controlling for age. Model 2 was fitted considering a possible interaction effect between delivery type and health insurance, while Model 3 was fitted including delivery type. Path analysis under structural equation modeling was used to ascertain the indirect effects of knowledge and attitude toward KMC on the practice of KMC.

### 2.5. Ethical Considerations

Ethical approval was obtained from the ethics committee of the Institute of Health Research of the University of Health and Allied Sciences (UHAS-REC A.7 [41] 24-25). The researchers adhere to a set of principles (voluntary participation, informed consent, anonymity, confidentiality, potential for harm, and results' communication) to ensure the study's integrity and the well-being of participants. This was done by diligently providing all potential participants with comprehensive information about the study's purpose, procedures, potential risks, and benefits. Before participation, written and verbal informed consent was obtained from each participant, ensuring their voluntary and uncoerced involvement. The entire consent process was documented transparently. Anonymity and confidentiality were ensured by allowing only the principal investigators to have access to the data collected on the Google Forms. Research assistants could only have the link that assisted them in administering the questionnaire. From the Google Form, the data was downloaded in Excel file format and transported to the statistical software for data analysis.

## 3. Results

The majority of the mothers were aged 18–35 years (59.8%), married (64.4%), Christians (95.7%), employed (89.4%), had tertiary education (83.3%), had a middle wealth index (87.4%), valid health insurance (88.9%), and Ewes (40.0%). Most of the newborns were perceived by the mothers to be healthy (96.5%), delivered through SVD (69.4%), and delivered at a government hospital (87.9%). More than one-third of the newborns weighed between 2000 and 2500 g, and 92.2% of the mothers perceived that newborns with preterm births require KMC.  Table 1 shows the demographic characteristics of mothers and children.

### 3.1. Predictors of Knowledge of KMC Among Mothers

The results showed that 46% of the mothers had adequate knowledge of KMC practice. From the bivariate analysis (Table 2), age, religion, wealth index, health insurance, health status of the baby, place of delivery, and weight of the newborn were associated with knowledge of KMC practice (*p* value < 0.05). In modeling the factors associated with knowledge of KMC among mothers, the model was significantly fitted with the likelihood ratio test (*p* value < 0.001), with Cox & Snell and Nagelkerke of 0.15 and 0.20, respectively.

The factors that predicted a woman having good knowledge of KMC were birthing a preterm or low birth weight baby, age between 18 and 35 years, being a Christian, having valid health insurance, and giving birth at a health facility. Mothers whose newborns weigh 1000–1499 g (aOR = 2.26, 95% CI: 1.08–4.88, *p* value = 0.033) and 2000–2500 g (aOR = 2.97, 95% CI: 1.45–6.32, *p* value = 0.004) were more likely to have good knowledge of KMC than mothers whose newborns weigh less than 1000 g. Similarly, Christians (aOR = 5.11, 95% CI: 1.26–28.25, *p* value = 0.035), those having valid health insurance (aOR = 6.75, 95% CI: 2.66–20.46, *p* value < 0.001), and those who delivered at the health centers (aOR = 14.86, 95% CI: 3.86–70.35, *p* value < 0.001) were more likely to have good knowledge of KMC than non-Christians, those with no health insurance, and those who delivered at government hospitals, respectively. However, young mothers aged 18–35 years (aOR = 0.45, 95% CI: 0.28–0.70, *p* value < 0.001) were less likely to have good knowledge of KMC than those older than 35. Table 2 shows the bivariate and multiple determinants of knowledge of KMC among mothers.

### 3.2. Predictors of Attitude Toward KMC Among Mothers

The overall attitude toward KMC practice among mothers was good (30.1%). From the bivariate analysis, the attitude of mothers toward KMC practice was significantly associated with education, ethnicity, health insurance, weight of newborn, and ANC follow-up (Table 3). The factors that predicted a woman having a positive attitude toward KMC were having a low birth weight baby and being an Akan. The model for predicting the attitude of mothers toward KMC was accurately fitted (*p* value < 0.001), with Cox & Snell and Nagelkerke pseudo-*R*-squared values of 0.13 and 0.18, respectively. Mothers whose newborns weighed 1000–1499 g (aOR = 2.78, 95% CI: 1.18–7.04, *p* value = 0.023), 1500–1999 g (aOR = 4.32, 95% CI: 1.78–11.28, *p* value = 0.002), and 2000–2500 g (aOR = 4.53, 95% CI: 1.99–11.25, *p* value = 0.001) were more likely to have good attitudes toward KMC practice. Akans (aOR = 0.51, 95% CI: 0.30–0.85, *p* value = 0.011) and other tribes (aOR = 0.14, 95% CI: 0.06–0.30, *p* value < 0.001) were less likely to have good attitudes toward KMC practice than Ewes.

### 3.3. Modeling the Predictors of Practice of KMC Among Mothers

The overall KMC practice among mothers was good (73.0%). From the bivariate analysis, KMC practice was significantly associated with health insurance, place of delivery, and weight of the newborn. The factors that significantly predicted a woman practicing KMC were the possession of health insurance, place of birth, and having preterm or low birth weight.  Table 4 shows the bivariate analysis of the factors influencing KMC practices among mothers.

Three models were fitted to ascertain the predictors of KMC practice among mothers. The first model was fitted with all possible factors of KMC practice that reported *p* values of at most 0.2 from the bivariate chi-square test of the association while controlling for age. Model 2 was fitted considering a possible interaction effect between delivery type and health insurance, while Model 3 was fitted including delivery type. The interaction model reported lower AIC, AICc, and BIC values of 446.4, 448, and 514.1 with Cox & Snell (0.12), Nagelkerke (0.17), and a significant likelihood ratio test (49.73, *p* value < 0.001). However, Model 1 reported higher AIC, AICc, and BIC values of 456.2, 457.5, and 515.9 with Cox & Snell (0.09), Nagelkerke (0.13), and a significant likelihood ratio test (35.89, *p* < 0.001). Similarly, Model 3 reported higher AIC, AICc, and BIC values of 455.9, 457.3, and 519.6 with Cox & Snell (0.09), Nagelkerke (0.13), and a significant likelihood ratio test (38.20, *p* value < 0.001). The interaction model was explained. The interactive model showed that the mode of delivery, having health insurance, birthing place, having a low birth weight, and those married significantly predicted the use of KMC.

From the interaction model, mothers who used the SVD type (aOR = 0.06, 95% CI: 0.01–0.28, *p* value = 0.001) are less likely to practice KMC than mothers who used the C/S delivery type. However, mothers who used the SVD type and had health insurance (aOR = 16.02, 95% CI: 3.13–94.95, *p* value = 0.001) are more likely to practice KMC than those without health insurance (Table 5). Mothers who delivered at a private hospital (aOR = 0.42, 95% CI: 0.18–0.97, *p* value = 0.039), as well as mothers with newborns with weights 1000–1499 g (aOR = 0.32, 95% CI: 0.13–0.72, *p* value = 0.008) and 2000–2500 g (aOR = 0.31, 95% CI: 0.13–0.70, *p* value = 0.006), were less likely to practice KMC. In addition, mothers who are not married (aOR = 1.93, 95% CI: 1.10–3.49, *p* value = 0.025) are more likely to practice KMC.

### 3.4. Indirect Effects of Knowledge and Attitude on KMC Practice

**Figure 1:**
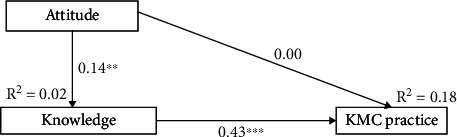
Mediation analysis of knowledge on the relationship between attitude and KMC practice (Model 1).

**Figure 2:**
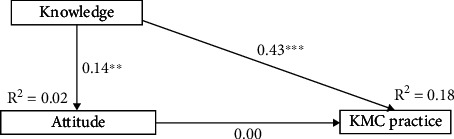
Mediation analysis of attitude on the relationship between knowledge and KMC practice (Model 2).

From Model 1, the attitude of mothers did not directly influence their perceived KMC practice (*p* value = 0.926); however, it indirectly influenced perceived KMC practice through the perceived knowledge of mothers (*p* value = 0.006). The perceived attitude of mothers positively influenced the perceived knowledge of mothers (*p* value = 0.004), while the perceived knowledge of mothers (*p* value < 0.001) positively influenced perceived KMC practice directly (Figure 1). However, from Model 2, the knowledge of mothers directly influenced their perceived KMC practice (*p* value < 0.001) but did not indirectly influence their perceived KMC practice through attitude (*p* value = 0.926) (Figure 2).

All the path models fitted accurately by reporting acceptable fit indices, such as CFI, GFI, SRMR, and RMSEA (Table 6).

### 3.5. Barriers to the Practice of KMC

**Figure 3:**
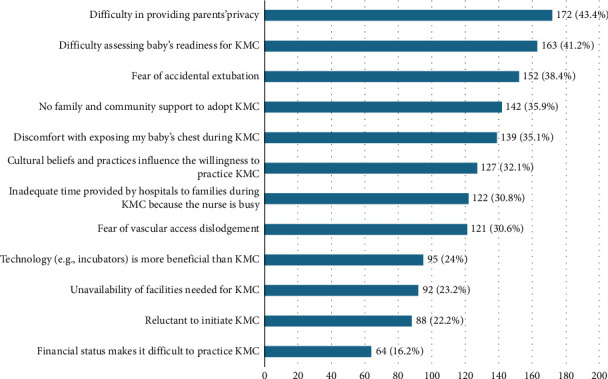
Barriers to KMC practice among mothers.

The barriers to KMC practice include difficulty in providing parents' privacy (43.4%), difficulty assessing the baby's readiness for KMC (41.2%), fear of accidental extubation (38.4%), no family and community support to adopt KMC (35.9%), discomfort with exposing the baby's chest during KMC (35.1%), cultural beliefs and practices influencing the willingness to practice KMC (32.1%), inadequate time provided by hospitals to families during KMC because the nurse is busy (30.8%), and fear of vascular access dislodgement (30.6%). Others include the perception of technology (e.g., incubators) being more beneficial than KMC (24%), the unavailability of facilities needed for KMC (23.2%), the reluctance to initiate KMC (22.2%), and lower financial status making it difficult to practice KMC (16.2%).  Figure 3 shows the barriers to KMC practices among mothers.

## 4. Discussion

The study contributes to the body of knowledge by showing the factors that influence the knowledge, attitude, and practice of KMC in a poor resource setting. Even though there have been global improvements in infant and child mortality rates, the case of developing countries has only been marginal [21, 22]. It was shown that having a low birth weight baby predicted women's higher knowledge, positive attitude, and consistent practice of KMC. Prioritizing the health of preterm and low birth weight infants warrants protection and ensuring adequate body temperature, appropriate breastfeeding techniques, effective bonding, and prevention of infections [4, 6, 8, 9]. This can be attained through utilizing KMC, especially in areas where health resources are limited [23]. Assessing the determinants for KMC in Africa is even more important because uptake is considerably low among preterm and low birth weight infants [24]. The factors that predicted a woman's attitudes toward KMC were having a low birth weight baby. One of the critical indicators for the use of KMC was noted as low birth weight or having a preterm baby [23–25]. Body temperature regulation is critical for the survival of preterm babies because of the limited adipose tissue they possess for thermoregulation [26, 27]. Parents must understand the specific needs of preterm and low birth weight infants to inculcate an attitude of using the body as a warm bag. For parents of these children to adopt this critical care measure, increasing maternal knowledge before or during ANC is critical. This will demand that mothers and caregivers understand the process of conducting and the benefits associated with KMC.

The factors that predict a woman's knowledge of KMC are birthing a preterm or low birth weight baby, age between 18 and 35 years, being a Christian, having health insurance, and giving birth at a health facility. Increasing maternal age [24] was found to be a significant determinant of mothers' knowledge of KMC. This may be correlated to experience, having a previous birth history, and frequent and consistent utilization of healthcare systems, including ANC [28]. Consequently, older women might have had childbirth experience, potentially increasing their knowledge of KMC. Previous studies further showed that women who attend higher-level (secondary and tertiary) health facilities appeared to have a higher knowledge of KMC compared to those who attended facilities at lower levels [23]. Therefore, health facilities must integrate KMC into the provision of maternal care services by providing a supporting environment, integrating into quality improvement, ensuring continuity of care, and promoting client-centered practices [17]. Health service providers must identify mothers who are at high risk for preterm births or low birth weight babies and implement measures to increase their knowledge by using tailored intervention methods, especially during the ANC or prebirth counseling. The factors that significantly predicted a woman practicing KMC were the possession of health insurance, place of birth, having a preterm or low birth weight, and being unmarried. Women who are elite and have financial resources are more likely to give birth within health facilities or to have attended ANC [29–31] and are likely to have learned about KMC during this period. Also, women who were not married showed an increased tendency to utilize KMC compared to those who were married. Reports about the influence of marital status on the utilization of KMC and practice appear to vary across geographical settings [9, 12, 32]. Unmarried women may have an increased propensity to practice KMC because they are the only care providers to the baby, compared to married people who may utilize spousal support [2, 7]. Encouraging maternal births within a health facility is critical for promoting effective health education and assessing the baby [33]. The adoption of KMC may be necessary to combat the negative emotional states often reported among mothers of preterm and low birth weight babies and a proactive attempt to improve parental self-image and increase responsibilities [34]. Women who delivered in a health facility had a higher chance of adopting and practicing KMC compared to those who delivered at home [24]. This is possibly attributable to the fact that women who delivered at healthcare facilities might have enjoyed facilitative support and had a comprehensive assessment of their babies, with the healthcare worker encouraging the adoption [35]. In addition, SVD mothers with health insurance were more likely to practice KMC compared to their counterparts. This may be as a result of the fact that women who deliver by SVD have shorter recovery time compared to those who deliver by CS and hence will be able to initiate KMC early [24]. Also, women possessing health insurance are more likely to have greater health risk awareness compared to those who do not have it [6, 30]. The interactive influence of the level of health awareness, education, and the faster recovery period may contribute to the higher chance of initiating and practicing KMC.

Diverse interrelated barriers were identified to influence a woman's likelihood of practicing KMC. As in previous studies [15], these factors encompass challenges associated with the healthcare system and its setup, as well as women- and family-related challenges and societal barriers, including cultural ones. This study showed that barriers to KMC practice include difficulty in providing parents' privacy, difficulty assessing the baby's readiness, fear of accidental extubation, no family and community support, discomfort with exposing the baby's chest, inadequate time provided by hospitals, lack of time by nurses, and fear of vascular access dislodgement. These factors were similarly identified in a systematic review to be critical for the implementation of KMC [15, 32]. In a low-infrastructure setting like the current study, local community knowledge, poor healthcare access, insufficient community-level amenities, inappropriate social support systems, and poor community infrastructure were identified as barriers to implementing KMC [15, 36]. The adoption of KMC for preterm and low birth weight usually requires concerted synergistic efforts to align with healthcare policy, eliminating barriers, facilitating resolution of challenges, supporting healthcare practitioners, and ensuring effective communication between parents and health workers. This can be achieved by effectively training mothers at the ANC on the methods and benefits of KMC. This is because mothers' understanding of KMC may enhance their likelihood of adoption and practice [18, 32]. Further studies must continue to identify the level of influence of each barrier to the eventual adoption of KMC among mothers.

This study is one of the first to be conducted in the Volta region of Ghana, addressing postnatal women regarding KMC. In addition, the study provides a divergent perspective on the concept of KMC as it provides insight into the women's knowledge, attitudes, and practices by highlighting the critical predictors. Identifying the level of knowledge, attitude, and practice of KMC is critical for developing comprehensive policy and guidelines for adoption to promote the practice. This study is not without some limitations. It is important to note that some limitations may include selection bias from convenience sampling. The impact of this limitation was probably limited due to the large sample size that was adopted. Also, generalizability to nontertiary settings should be done with caution. In Ghana, health services and baby care can be obtained from primary to tertiary level health facilities. But this study was basically confined to a tertiary healthcare facility, which is usually a referral setting. In addition, this study collected self-reported data, leaving it with the risk of self-reporting bias and social desirability bias.

## 5. Conclusion

This study predicted the factors that influence the knowledge, attitude, and practice of KMC in low-resource settings. We identified that a woman having a preterm baby predicted women's higher knowledge, positive attitude, and consistent practice of KMC. In addition, given that numerous factors influence the practice (SVD, having health insurance, not birthing in a private facility, and having a normal birth weight baby) of KMC, promoting health insurance registration, increasing pregnancy safety leading to SVD, and fostering normal birth weight births can limit the need to use KMC. However, given the positive benefits of KMC, ensuring a positive attitude among community members is crucial to its adoption, should it be needed. Furthermore, health service providers must identify mothers who are at high risk for low birth weight babies and implement measures to increase their knowledge by using tailored interventions, especially during the ANC or prebirth counseling. Tailored interventions must target mothers with preterm births or at risk of preterm births, together with their families. Pregnant women must be empowered during the ANC period to gain adequate knowledge, positive attitudes, and effective skills to practice KMC.

## Figures and Tables

**Table 1:** Demographic characteristics of mothers and children.

**Variables**	**Frequency**	**Percentage**
Age		
18–35	237	59.8
> 35	159	40.2
Marital status		
Unmarried	141	35.6
Married	255	64.4
Education		
Below tertiary	66	16.7
Tertiary	330	83.3
Ethnicity		
Ewe	158	39.9
Akan	151	38.1
Others	87	22
Religion		
Christian	379	95.7
Non-Christian	17	4.3
Employment		
Unemployed	42	10.6
Employed	354	89.4
Wealth index		
Poor	12	3
Middle	346	87.4
Rich	38	9.6
Health insurance		
Yes	352	88.9
No	44	11.1
Sex of infant		
Male	201	50.8
Female	195	49.2
Perceived health status of the baby		
Healthy	382	96.5
Sick	14	3.5
Delivery type		
SVD	275	69.4
C/S	121	30.6
Place of delivery		
Government hospital	348	87.9
Health center	15	3.8
Private hospital	33	8.3
Weight of newborn		
< 1000 g	60	15.2
1000–1499 g	111	28
1500–1999 g	88	22.2
2000–2500 g	137	34.6
ANC follow-up		
Yes	373	94.2
No	23	5.8
Perceived newborns that require KMC		
Preterm	365	92.2
Postterm/baby greater than 4 kg	31	7.8

**Table 2:** Bivariate and multiple determinants of knowledge of KMC among mothers.

**Variables**	**Knowledge of KMC practice**			
	**Good**	**Poor**	**cOR (95% CI), ** **p** ** value**	**aOR (95% CI), ** **p** ** value**
Age				
18–35	95 (40.1)	142 (59.9)	0.53 (0.35, 79), 0.002	0.45 (0.28, 0.70), < 0.001
> 35	89 (56)	70 (44)	Ref	Ref
Marital status				
Unmarried	68 (48.2)	73 (51.8)	1.12 (0.74, 1.69), 0.601	
Married	116 (45.5)	139 (54.5)	Ref	
Education				
Below tertiary	25 (37.9)	41 (62.1)	0.66 (0.38, 1.13), 0.126	1.03 (0.52, 2.02), 0.939
Tertiary	159 (48.2)	171 (51.8)	Ref	Ref
Ethnicity				
Ewe	76 (48.1)	82 (51.9)		
Akan	75 (49.7)	76 (50.3)	1.07 (0.68, 1.66), 0.783	0.98 (0.59, 1.64), 0.941
Others	33 (37.9)	54 (62.1)	0.66 (0.39, 1.12), 0.125	0.73 (0.39, 1.34), 0.312
Religion				
Christian	181 (47.8)	198 (52.2)	4.27 (1.21, 15.09), 0.015	5.11 (1.26, 28.25), 0.035
Non-Christian	3 (17.6)	14 (82.4)	Ref	Ref
Employment				
Unemployed	20 (47.6)	22 (52.4)		
Employed	164 (46.3)	190 (53.7)	0.95 (0.50, 1.80), 0.874	Ref
Wealth index				
Poor	0 (0)	12 (100)	< 0.001	
Middle	159 (46)	187 (54)	0.44 (0.22, 0.89), 0.020	
Rich	25 (65.8)	13 (34.2)	Ref	
Health insurance				
Yes	176 (50)	176 (50)	4.5 (2.03, 9.96), < 0.001	6.75 (2.66, 20.46), < 0.001
No	8 (18.2)	36 (81.8)	Ref	Ref
Sex of the infant				
Male	86 (42.8)	115 (57.2)	0.74 (0.50, 1.10), 0.136	0.7 (0.44, 1.10), 0.120
Female	98 (50.3)	97 (49.7)	Ref	Ref
Health status of the baby				
Healthy	184 (48.2)	198 (51.8)	< 0.001	
Sick	0 (0)	14 (100)		
Delivery type				
SVD	128 (46.5)	147 (53.5)	1.01 (0.66, 1.55), 0.961	
C/S	56 (46.3)	65 (53.7)	Ref	
Place of delivery				
Government hospital	165 (47.4)	183 (52.6)	Ref	Ref
Health center	11 (73.3)	4 (26.7)	3.05 (0.95, 9.76), 0.049	14.86 (3.86, 70.35), < 0.001
Private hospital	8 (24.2)	25 (75.8)	0.36 (0.16, 0.81), 0.011	0.6 (0.23, 1.48), 0.275
Weight of the newborn				
< 1000 g	19 (31.7)	41 (68.3)	Ref	Ref
1000–1499 g	52 (46.8)	59 (53.2)	1.90 (0.98, 3.68), 0.055	2.26 (1.08, 4.88), 0.033
1500–1999 g	40 (45.5)	48 (54.5)	1.80 (0.91, 3.57), 0.093	2.2 (1.00, 4.97), 0.052
2000–2500 g	73 (53.3)	64 (46.7)	2.46 (1.30, 4.66), 0.005	2.97 (1.45, 6.32), 0.004
ANC follow-up				
Yes	174 (46.6)	199 (53.4)	1.14 (0.49, 2.66), 0.767	
No	10 (43.5)	13 (56.5)	Ref	

**Table 3:** Bivariate and multiple determinants of attitude toward KMC practice among mothers.

**Variables**	**Attitude toward KMC practice**			
	**Good**	**Poor**	**cOR (95% CI), ** **p** ** value**	**aOR (95% CI), ** **p** ** value**
Age				
18–35	69 (29.1)	168 (70.9)	0.90 (0.58, 1.39), 0.620	1.00 (0.62, 1.62), 0.993
> 35	50 (31.4)	109 (68.6)	Ref	Ref
Marital status				
Unmarried	38 (27)	103 (73)	0.79 (0.50, 1.25), 0.317	
Married	81 (31.8)	174 (68.2)	Ref	
Education				
Below tertiary	11 (16.7)	55 (83.3)	0.41 (0.21, 0.82), 0.009	0.66 (0.28, 1.48), 0.323
Tertiary	108 (32.7)	222 (67.3)		
Ethnicity				
Ewe	61 (38.6)	97 (61.4)		
Akan	49 (32.5)	102 (67.5)	0.76 (0.48, 1.22), 0.258	0.51 (0.30, 0.85), 0.011
Others	9 (10.3)	78 (89.7)	0.18 (0.09, 0.39), < 0.001	0.14 (0.06, 0.30), < 0.001
Religion				
Christian	114 (30.1)	265 (69.9)	1.03 (0.36, 3.00), 0.953	
Non-Christian	5 (29.4)	12 (70.6)	Ref	
Employment				
Unemployed	8 (19)	34 (81)	Ref	Ref
Employed	111 (31.4)	243 (68.6)	1.94 (0.87, 4.33), 0.100	1.03 (0.40, 2.81), 0.960
Wealth index				
Poor	2 (16.7)	10 (83.3)	0.39 (0.07, 2.02), 0.248	
Middle	104 (30.1)	242 (69.9)	0.83 (0.41, 1.68), 0.598	
Rich	13 (34.2)	25 (65.8)	Ref	
Health insurance				
Yes	119 (33.8)	233 (66.2)	< 0.001	
No	0 (0)	44 (100)		
Sex of infant				
Male	53 (26.4)	148 (73.6)	0.70 (0.46, 1.09), 0.105	0.67 (0.42, 1.08), 0.102
Female	66 (33.8)	129 (66.2)	Ref	Ref
Health status of the baby				
Healthy	117 (30.6)	265 (69.4)	Ref	
Sick	2 (14.3)	12 (85.7)	0.38 (0.08, 1.71), 0.245	
Delivery type				
SVD	85 (30.9)	190 (69.1)	1.15 (0.71, 1.84), 0.574	
C/S	34 (28.1)	87 (71.9)	Ref	
Place of delivery				
Government hospital	105 (30.2)	243 (69.8)	Ref	
Health center	4 (26.7)	11 (73.3)	0.84 (0.26, 2.70), 0.789	
Private hospital	10 (30.3)	23 (69.7)	1.01 (0.46, 2.19), 0.988	
Weight of newborn				
< 1000 g	9 (15)	51 (85)	Ref	Ref
1000–1499 g	26 (23.4)	85 (76.6)	1.73 (0.75, 3.99), 0.193	2.78 (1.18, 7.04), 0.023
1500–1999 g	34 (38.6)	54 (61.4)	3.57 (1.56, 8.17), 0.002	4.32 (1.78, 11.28), 0.002
2000–2500 g	50 (36.5)	87 (63.5)	3.26 (1.48, 7.17), 0.002	4.53 (1.99, 11.25), 0.001
ANC follow-up				
Yes	117 (31.4)	256 (68.6)	4.8 (1.11, 20.80), 0.021	3.09 (0.80, 20.43), 0.152
No	2 (8.7)	21 (91.3)	Ref	Ref
Perceived newborns that require KMC				
Preterm	111 (30.4)	254 (69.6)	1.26 (0.55, 2.90), 0.591	
Postterm/baby greater than 4 kg	8 (25.8)	23 (74.2)	Ref	

**Table 4:** Bivariate analysis of factors influencing KMC practice among mothers.

**Variables**	**KMC practice**		
	**Yes**	**No**	**cOR (95% CI), ** **p** ** value**
Age			
18–35	172 (72.6)	65 (27.4)	0.95 (0.60, 1.50), 0.824
> 35	117 (73.6)	42 (26.4)	Ref
Marital status			
Unmarried	110 (78)	31 (22)	1.51 (0.93, 2.44), 0.093
Married	179 (70.2)	76 (29.8)	Ref
Education			
Below tertiary	42 (63.6)	24 (36.4)	0.59 (0.34, 1.03), 0.061
Tertiary	247 (74.8)	83 (25.2)	Ref
Ethnicity			
Ewe	114 (72.2)	44 (27.8)	Ref
Akan	114 (75.5)	37 (24.5)	1.19 (0.72, 1.98), 0.504
Others	61 (70.1)	26 (29.9)	0.91 (0.51, 1.61), 0.736
Religion			
Christian	279 (73.6)	100 (26.4)	1.95 (0.72, 5.27), 0.261
Non-Christian	10 (58.8)	7 (41.2)	Ref
Employment			
Unemployed	32 (76.2)	10 (23.8)	Ref
Employed	257 (72.6)	97 (27.4)	0.83 (0.39, 1.75), 0.620
Wealth index			
Poor	10 (83.3)	2 (16.7)	0.94 (0.16, 5.40), 1.000
Middle	247 (71.4)	99 (28.6)	0.47 (0.19, 1.15), 0.092
Rich	32 (84.2)	6 (15.8)	Ref
Health insurance			
Yes	263 (74.7)	89 (25.3)	2.05 (1.07, 3.91), 0.028
No	26 (59.1)	18 (40.9)	Ref
Sex of infant			
Male	150 (74.6)	51 (25.4)	1.19 (0.76, 1.85), 0.454
Female	139 (71.3)	56 (28.7)	Ref
Health status of the baby			
Healthy	279 (73)	103 (27)	1.08 (0.33, 3.53), 1.000
Sick	10 (71.4)	4 (28.6)	Ref
Delivery type			
SVD	198 (72)	77 (28)	0.85 (0.52, 1.38), 0.508
C/S	91 (75.2)	30 (24.8)	Ref
Place of delivery			
Government hospital	261 (75)	87 (25)	Ref
Health center	10 (66.7)	5 (33.3)	0.67 (0.22, 2.00), 0.468
Private hospital	18 (54.5)	15 (45.5)	0.40 (0.19, 0.83), 0.011
Weight of newborn			
< 1000 g	50 (83.3)	10 (16.7)	Ref
1000–1499 g	72 (64.9)	39 (35.1)	0.37 (0.17, 0.81), 0.011
1500–1999 g	71 (80.7)	17 (19.3)	0.84 (0.35, 1.98), 0.682
2000–2500 g	96 (70.1)	41 (29.9)	0.47 (0.22, 1.01), 0.051
ANC follow-up			
Yes	275 (73.7)	98 (26.3)	1.80 (0.76, 4.30), 0.178
No	14 (60.9)	9 (39.1)	Ref
Perceived newborns that require KMC			
Preterm	270 (74)	95 (26)	1.80 (0.84, 3.84), 0.127
Postterm/baby greater than 4 kg	19 (61.3)	12 (38.7)	Ref

**Table 5:** Models for determinants of KMC practice among mothers.

**Predictors**	**Model 1**	**Model 2**	**Model 3**
	**aOR (95% CI), ** **p** ** value**	**aOR (95% CI), ** **p** ** value**	**aOR (95% CI), ** **p** ** value**
Wealth index [rich]	Ref	Ref	Ref
Wealth index [middle]	0.38 (0.13, 0.97), 0.059	0.39 (0.13, 1.04), 0.077	0.37 (0.12, 0.95), 0.054
Wealth index [poor]	0.88 (0.15, 7.11), 0.894	0.98 (0.16, 8.08), 0.980	0.88 (0.15, 7.11), 0.896
Marital status [married]	Ref	Ref	Ref
Marital status [unmarried]	1.89 (1.09, 3.38), 0.026	1.93 (1.10, 3.49), 0.025	1.92 (1.11, 3.43), 0.023
Weight of newborn [< 1000 g]	Ref	Ref	Ref
Weight of newborn [1000–1499 g]	0.31 (0.13, 0.69), 0.006	0.32 (0.13, 0.72), 0.008	0.3 (0.12, 0.66), 0.004
Weight of newborn [1500–1999 g]	0.75 (0.29, 1.87), 0.547	0.58 (0.22, 1.47), 0.258	0.71 (0.27, 1.78), 0.478
Weight of newborn [2000–2500 g]	0.35 (0.15, 0.78), 0.014	0.31 (0.13, 0.70), 0.006	0.35 (0.14, 0.77), 0.012
ANC follow-up [no]			
ANC follow-up [yes]	1.87 (0.65, 5.38), 0.243	2.23 (0.77, 6.41), 0.133	1.99 (0.69, 5.71), 0.198
Perceived newborns that require KMC [postterm/baby greater than 4 kg]	Ref	Ref	Ref
Perceived newborns that require KMC [preterm]	2.18 (0.87, 5.28), 0.088	2.36 (0.94, 5.75), 0.062	2.09 (0.83, 5.08), 0.108
Education [tertiary]	Ref	Ref	Ref
Education [below tertiary]	0.87 (0.44, 1.75), 0.686	0.62 (0.31, 1.28), 0.191	0.84 (0.43, 1.70), 0.630
Age [> 35]	Ref	Ref	Ref
Age [18–35]	0.89 (0.54, 1.45), 0.631	0.86 (0.52, 1.43), 0.570	0.9 (0.55, 1.48), 0.690
Delivery type [C/S]	Ref	Ref	Ref
Delivery type [SVD]		0.06 (0.01, 0.28), 0.001	0.66 (0.38, 1.13), 0.135
Health insurance [no]	Ref	Ref	Ref
Health insurance [yes]	1.95 (0.91, 4.12), 0.080	0.49 (0.12, 1.65), 0.271	2.22 (1.01, 4.81), 0.043
Place of delivery [government hospital]	Ref	Ref	Ref
Place of delivery [health center]	0.69 (0.21, 2.52), 0.547	0.66 (0.19, 2.46), 0.509	0.69 (0.21, 2.50), 0.546
Place of delivery [private hospital]	0.44 (0.19, 0.99), 0.044	0.42 (0.18, 0.97), 0.039	0.44 (0.19, 1.00), 0.046
Delivery type [SVD] × health insurance [yes]		16.02 (3.13, 94.95), 0.001	

**Table 6:** Path models for indirect effects of knowledge and attitude on KMC practice.

**Models**	**Paths**	**Through**	**Coefficient**	**p** ** value**
Model 1 (CFI = 1.000, GFI = 1.000, SRMR = 0.000, RMSEA = 0.00)				
H1	Attitude → KMC practice		0.00	0.926
H2	Attitude → knowledge		0.14	0.004
H3	Knowledge → KMC practice		0.43	< 0.001
H4	Attitude → KMC practice	Knowledge	0.06	0.006
Model 2 (CFI = 1.000, GFI = 1.000, SRMR = 0.000, RMSEA = 0.000)				
H1	Knowledge → KMC practice		0.43	< 0.001
H2	Knowledge → attitude		0.14	0.004
H3	Attitude → KMC practice		0.00	0.926
H4	Knowledge → KMC practice	Attitude	0.00	0.926

## Data Availability

The data that support the findings of this study are available from the corresponding author upon reasonable request.
